# Distribution and characteristics of ceftiofur-resistant *Escherichia coli* throughout the broiler production chain in Korea

**DOI:** 10.1186/s13567-025-01668-9

**Published:** 2026-03-24

**Authors:** Jae Kyung Lee, Bo-Youn Moon, Suk-Kyung Lim, Young Ju Lee

**Affiliations:** 1 College of Veterinary Medicine & Institute for Veterinary Biomedical Science, Kyungpook National University, Daegu, 41566 Republic of Korea; 2 Bacterial Disease Division, Animal and Plant Quarantine Agency, Gimcheon-Si, Gyeongsangbuk-Do 39660 Republic of Korea

**Keywords:** Ceftiofur, ceftiofur-resistant* Escherichia coli*, integrated broiler operation

## Abstract

Ceftiofur, a third-generation cephalosporin, is classified as a critically important antimicrobial in both human and veterinary medicine, but its use in poultry has raised public health concerns due to an increase in resistant pathogens. This study comprehensively investigated the distribution and characteristics of ceftiofur-resistant *Escherichia coli* (*E. coli*) across the production stages of three major integrated broiler operations in Korea. Although ceftiofur was only used via subcutaneous injection in day-old chicks at breeder hatcheries and not in other stages of integrated broiler production, ceftiofur-resistant *E. coli* were detected throughout all stages of integrated broiler production. The prevalence of ceftiofur-resistant *E. coli* isolates was generally greater in fluff and eggshells from breeder hatcheries (15.4%–47.4%) and commercial broiler hatcheries (31.6%–51.7%) than in environmental dust from broiler breeder farms (11.5%–38.5%) and commercial broiler farms (8.7%–28.9%). Notably, resistant *E. coli* in slaughterhouse carcasses correlated with a high prevalence in farm dust at the time of flock depletion. Compared with the susceptible isolates (0.0%–75.7%), the ceftiofur-resistant isolates (20.5%–100.0%) presented significantly greater coresistance to other antimicrobials (*p* < 0.05). Among the 239 resistant isolates, 92.9% harboured at least one β-lactamase gene, predominantly *bla*_CTX-M-55_, *bla*_TEM-1_, or *bla*_CMY-2_ (11.3%–58.9%). All resistant isolates carrying β-lactamase genes also had plasmid replicons, mainly IncFII and IncFIB (41.7%–60.5%). These findings suggest that voluntary or mandatory prohibition of the off-label use of ceftiofur in breeder hatcheries is necessary to prevent the dissemination of ceftiofur-resistant *E. coli* throughout the stages of integrated broiler production.

## Introduction

Ceftiofur is a 3^rd^-generation cephalosporin developed exclusively for veterinary use [1], and 3^rd^-generation cephalosporins are classified as critically important antimicrobial agents in human and veterinary medicine because they represent one of the few remaining treatment options for infections caused by multidrug-resistant (MDR) Enterobacteriaceae [2, 3]. In particular, ceftiofur is structurally similar to ceftriaxone and cefotaxime, which are 3^rd^-generation cephalosporins widely used in human medicine, and it shares the same mechanism of action [1]; therefore, infections caused by ceftiofur-resistant bacteria can further limit the options available for treating bacterial infectious diseases in humans.

Many researchers have reported that the use of ceftiofur in poultry production is responsible for the increase in resistant *Escherichia coli* and *Salmonella* spp. in many countries [4–6]. In particular, Saraiva et al., reported that the common practice of administering ceftiofur to day-one chicks increases the short-term shedding of extended-spectrum β-lactamase (ESBL)-producing *E. coli* [7]. ESBLs are enzymes that make bacteria resistant to many β-lactam antimicrobial agents, including penicillins, cephalosporins, and monobactams [8], and ESBL genes are often located on highly transmissible mobile genetic elements such as plasmids, transposons, and integrons [9, 10]. Therefore, ceftiofur has been coadministered with Marek’s disease vaccine in poultry hatcheries through *in ovo* injection when embryonic eggs are 17–18 days or via subcutaneous injection in day-old chicks in the United States, the European Union, Japan, and Canada, but it is now even prohibited for off-label use [11–14].

In Korea, ceftiofur is not approved for use in poultry, but unlike other countries, ceftiofur is still used off-label in combination with Marek’s disease vaccine via subcutaneous injection into a day-old chick in poultry hatcheries to prevent early mortality caused by *E. coli* infections. According to the Korean Veterinary Antimicrobial Resistance Monitoring System (KVARMS), the prevalence of ceftiofur- or cefotaxime-resistant *E. coli* isolates from broiler carcasses continuously increased from 11.0% in 2021 to 19.6% in 2023 [15]. In Korea, integrated broiler operation, which is a vertical system that includes breeder hatcheries, breeder farms, broiler hatcheries, broiler farms, slaughterhouses, feed mills, and processing plants, accounts for approximately 96.4% of broiler meat production [16]. These broiler operations control and operate through all stages of the broiler industry, such as breeder flock management, hatchery operation, biosecurity management, feed management, broiler slaughter, and retail distribution [17], and the top five integrated broiler operations account for 51.4% of the market share [16]. Therefore, the off-label use of ceftiofur in hatcheries may lead to the vertical transmission of ceftiofur-resistant *E. coli* across stages of integrated broiler production in Korea. Although the characteristics of ceftiofur-resistant *E. coli* from commercial broiler farms and chicken meat have been consistently reported in Korea [18–20], there has been limited research on the prevalence and dissemination of ceftiofur-resistant *E. coli* isolates across the stages of integrated broiler production. Therefore, this study aimed to comprehensively analyse the distribution and characteristics of ceftiofur-resistant *E. coli* throughout the broiler production stages of three major integrated broiler operations among the top five in Korea and to provide a basis for controlling its spread.

## Materials and methods

### Sampling stage

**Figure 1:**
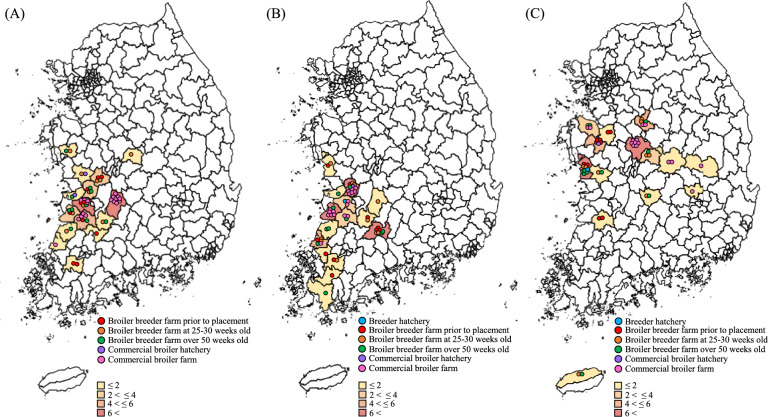
**Distribution of stages of integrated broiler production by operations A (A), B (B), and C (C)**. Sampling was conducted at different times on the same commercial broiler farms, but different breeder farms were selected for each operation on the basis of the appropriate sampling time point.

In 2024, among the production stages of three vertically integrated broiler operations, sampling was conducted at breeder hatcheries, breeder farms, broiler hatcheries, and broiler farms. Although the three integrated broiler operations are among the top five integrated broiler operations in Korea, only two operations have their own breeder hatcheries, whereas one operation conducts contract hatching. However, all three operations have two commercial broiler hatcheries each. Therefore, sampling was conducted in two breeder hatcheries and a total of four commercial broiler hatcheries (Table 1). Moreover, a total of 90 broiler breeder farms (30 farms from each operation) and 51 commercial broiler farms (15–18 farms from each operation) were randomly selected for sampling (Figure 1). Sampling was conducted at different times (prior to placement and at the time of depletion) on the same commercial broiler farms, but different farms were selected for each operation on the basis of the appropriate sampling time point (prior to placement, at 25–30 weeks of age, and over 50 weeks of age). A situational analysis of ceftiofur use at the production stage was conducted through a questionnaire-based survey to collect information from hatcheries or farm owners.

**Table 1:** **Hatchery capacity in three integrated broiler operations**

Hatchery capacity	Breeder hatchery			Commercial broiler hatchery		
	Operation A	Operation B	Operation C	Operation A	Operation B	Operation C
No. of hatchery owned	0^1^	1	1	2	2	2
No. of hatchery tested	0	1	1	2	1	1
No. of setter per tested hatchery	–	15	12	52/90	42	36
No. of hatcher per tested hatchery	–	12	8	24/40	42	21
Hatching capacity^2^	–	460 000	300 000	1 500 000/2 200 000	1 600 000	1 600 000

^1^Operation A did not have a breeder hatchery and carried out contract hatching

^2^No. of eggs hatched per week per hatcher

### Sample collection

The fluff and eggshells were sampled from breeder and commercial broiler hatcheries by modifying the process for the collection of fluff samples of the standards set by the National Poultry Improvement Plan (NPIP) [21]. Approximately 10 g of fluff and 100 g of eggshell from the floor or tray after hatching were obtained from five hatchers per hatchery and were collected twice at different times for the same hatchery. The environmental dust was sampled from up to three houses per farm at different rearing times. Specifically, dust was collected from houses prior to placement, at 25–30 weeks of age, and over 50 weeks of age for breeder farms and prior to placement and at the time of depletion for commercial broiler farms. In accordance with the standards set by the NPIP [21], approximately 10 g of environmental dust was obtained by swabbing 15 different spots per house with sterile surgical gauze moistened with buffered peptone water (BPW, Difco, Sparks). All the samples were placed in sterile bags and transported to the laboratory at 4 °C.

### Bacterial identification

Bacterial isolation from fluff, eggshell, and environmental dust samples was performed according to the Ministry of Food and Drug Safety guidelines [22]. Briefly, each sample was inoculated into BPW at a ratio of 1:10. After incubation at 37 °C ± 2 °C for 18–24 h**,** 1 mL of preenriched BPW was inoculated into 9 mL of mEC broth supplemented with novobiocin (Merck, Darmstadt, Germany) and incubated at 44 °C ± 1 °C for 22–26 h. The cultured mEC broth was streaked onto MacConkey agar (BD Biosciences, Sparks, MD, USA). At least two presumptive *E. coli* colonies were selected from MacConkey agar plates and confirmed by polymerase chain reaction (PCR), as previously described [23]. If some isolates from different hatchers or houses within the same hatchery or farm presented the same antimicrobial susceptibility patterns, only one isolate was randomly selected.

### Antimicrobial susceptibility testing

Antimicrobial susceptibility testing was conducted in accordance with the guidelines of the Clinical and Laboratory Standards Institute [24]. All *E. coli* isolates were investigated for antimicrobial resistance using the disk diffusion method with the following antimicrobial disks (BD Biosciences, Sparks, MD): ampicillin (10 μg), amoxicillin-clavulanate (20/10 μg), cefoxitin (30 μg), cefotaxime (30 μg), ceftazidime (30 μg), cefepime (30 μg), chloramphenicol (30 μg), ciprofloxacin (5 μg), gentamicin (10 μg), tetracycline (30 μg), and trimethoprim-sulfamethoxazole (1.25/23.75 μg). Moreover, minimum inhibitory concentrations (MICs) for cefotaxime, ceftazidime, ceftiofur, and cefepime were determined at concentrations ranging from 0.5 to 512 μg/mL using standard agar dilution methods with Mueller‒Hinton agar (BD Biosciences), following CLSI guidelines (CLSI, 2024). MDR was defined as acquired resistance to at least one agent in three or more antimicrobial classes [25]. Additionally, the prevalence of resistance to cefotaxime, a 3^rd^-generation cephalosporin, in *E. coli* isolates from carcasses in slaughterhouses of the same three integrated broiler operations was provided by the KVARMS of the Animal and Plant Quarantine Agency (APQA), which officially conducts antimicrobial resistance testing for livestock.

### Molecular analysis of antimicrobial resistance genes

The presence of β‑lactamase genes [*bla*_TEM_, *bla*_SHV_, *bla*_OXA_*, bla*_CTX-M_, and plasmid-mediated AmpC (pAmpC)] was confirmed by PCR using the primers listed in Table 2. Moreover, the PCR products of the β‑lactamase genes were sequenced using an automatic sequencer (Cosmogenetech, Daejeon, Korea), and the DNA sequence data were compared with those in the GenBank nucleotide database using the Basic Local Alignment Search Tool program available through the National Center for Biotechnology Information website.

**Table 2:** **Primers used in this study**

Target	Sequence (5' → 3')	Size (bp)	Reference
β-lactamase gene			
CTX-M group I	F: GACGATGTCACTGGCTGAGC R: AGCCGCCGACGCTAATACA	499	[68]
CTX-M group II	F: GCGACCTGGTTAACTACAATCC R: CGGTAGTATTGCCCTTAAGCC	351	[68]
CTX-M group III	F: CGCTTTGCCATGTGCAGCACC R: GCTCAGTACGATCGAGCC	307	[68]
CTX-M group IV	F: GCTGGAGAAAAGCAGCGGAG R: GTAAGCTGACGCAACGTCTG	474	[68]
OXA	F: TTCAAGCCAAAGGCACGATAG R: TCCGAGTTGACTGCCGGGTTG	702	[69]
SHV	F: CACTCAAGGATGTATTGTG R: TTAGCGTTGCCAGTGCTCG	885	[69]
TEM	F: CATTTCCGTGTCGCCCTTATTC R: CGTTCATCCATAGTTGCCTGAC	800	[70]
pAmpC gene			
ACCM	F: AACAGCCTCAGCAGCCGGTTA R: TTCGCCGCAATCATCCCTAGC	346	[71]
CITM	F: TGGCCAGAACTGACAGGCAAA R: TTTCTCCTGAACGTGGCTGGC	462	[71]
DHAM	F: AACTTTCACAGGTGTGCTGGGT R: CCGTACGCATACTGGCTTTGC	405	[71]
EBCM	F: TCGGTAAAGCCGATGTTGCGG R: CTTCCACTGCGGCTGCCAGTT	302	[71]
FOXM	F: AACATGGGGTATCAGGGAGATG R: CAAAGCGCGTAACCGGATTGG	190	[71]
MOXM	F: GCTGCTCAAGGAGCACAGGAT R: CACATTGACATAGGTGTGGTGC	520	[71]

### Plasmid replicon typing

The plasmid incompatibility (Inc) groups of ceftiofur-resistant *E. coli* isolates harboring β‑lactamase were determined using PCR-based replicon typing with 18 pairs of primers, as previously described by Carattoli et al. [26].

### Statistical analysis

Statistical analyses were conducted using the Statistical Package for the Social Sciences (SPSS) v. 29 (IBM Corp., Armonk, NY, USA). Pearson’s chi-square test with Bonferroni correction was also performed. Differences were considered significant at *p* < 0.05.

## Results

### Ceftiofur use by stages of integrated broiler production

The results of the analysis of ceftiofur use in the three integrated broiler production stages are shown in Table 3. Ceftiofur was only coadministered with Marek’s disease vaccine via subcutaneous injection to day-old chicks in all breeder hatcheries, but ceftiofur was never used for therapeutic or prophylactic purposes in broiler breeder farms, commercial broiler hatcheries, or commercial broiler farms. In addition, *in ovo* injection of Marek’s disease vaccine was not performed at any stage of the three integrated broiler operations.

**Table 3:** **Ceftiofur use in the production stages of three integrated broiler operations**

Production stage	Administration route	No. of hatcheries or farms administered ceftiofur/No. of hatcheries or farms tested by operation (%)			
		A	B	C	Total
Breeder hatchery	*In-ovo* injection	NT^1^	0/2 (0.0)	0/2 (0.0)	0/4 (0.0)
	Subcutaneous injection^2^	NT	2/2 (100.0)	2/2 (100.0)	4/4 (100.0)
Broiler breeder farm	Subcutaneous injection	0/10 (0.0)	0/10 (0.0)	0/10 (0.0)	0/30 (0.0)
Commercial broiler hatchery	*In-ovo* injection	0/4 (0.0)	0/2 (0.0)	0/2 (0.0)	0/8 (0.0)
	Subcutaneous injection	0/4 (0.0)	0/2 (0.0)	0/2 (0.0)	0/8 (0.0)
Commercial broiler farm	Subcutaneous injection	0/18 (0.0)	0/18 (0.0)	0/15 (0.0)	0/51 (0.0)

^1^NT, not tested

^2^Ceftiofur was coadministered with Marek’s disease vaccine in all tested breeder hatcheries

### Prevalence of ceftiofur-resistant *E. coli* isolates by stage of integrated broiler production

**Figure 2:**
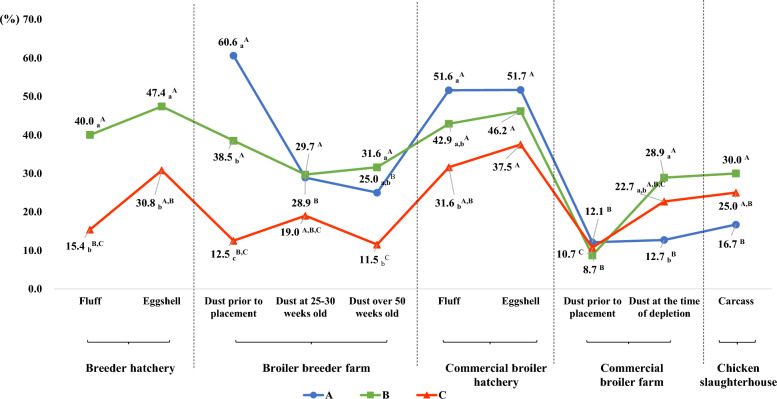
**Prevalence of ceftiofur-resistant E. coli isolates by production stage in three integrated broiler operations**. As operation A does not involve a breeder hatchery, it was not tested. All the *E. coli* isolates were tested for resistance to ceftiofur, but the *E. coli* strains isolated from the carcasses of the slaughterhouses were tested for resistance to cefotaxime. The uppercase letters (^A, B^) represent significant differences among sample types in each operation, whereas the lowercase letters (_a, b_) represent significant differences among operations in each sample type (*p* < 0.05).

The distribution of ceftiofur-resistant *E. coli* isolates by stage of integrated broiler production is shown in Figure 2. The prevalence of ceftiofur-resistant *E. coli* isolates significantly differed among the broiler production stages (*p* < 0.05). The prevalence of ceftiofur-resistant *E. coli* isolates in dust prior to placement on broiler breeder farms in operation A, which practice contract hatching, was 60.6%, which was the highest among all broiler production stages. However, in general, the prevalence of fluff (15.4%–40.0%) and eggshell (30.8%–47.4%) from breeder hatcheries and of fluff (31.6%–51.6%) and eggshell (37.5%–51.7%) from commercial broiler hatcheries was greater than that of dust from broiler breeder farms (11.5%–38.5%, except for operation A) and commercial broiler farms (8.7%–28.9%). Moreover, the prevalence of ceftiofur-resistant *E. coli* isolates also significantly differed among the three integrated broiler operations (*p* < 0.05). In particular, the prevalence of ceftiofur-resistant *E. coli* isolates in breeder hatcheries, broiler breeder farms, and commercial broiler hatcheries was significantly lower in operation C (11.5%–37.5%) than in operations A (25.0%–60.6%) and B (24.4%–48.3%) (*p* < 0.05). However, the prevalence of dust at the time of depletion in commercial broiler farms was significantly greater in operations B (28.9%) and C (22.7%) than in operation A (12.7%), which resulted in a significantly greater prevalence of cefotaxime-resistant *E. coli* isolates in carcasses from operations B (30.0%) and C (25.0%) than in those from operation A (16.7%) (*p* < 0.05).

### Distribution of MICs of *E. coli* isolates by stages of integrated broiler production

The distribution of MICs for 3^rd^^−^ and 4^th^-generation cephalosporins in *E. coli* isolates by stage of integrated broiler production is shown in Table 4. In general, the MIC_50_ values for 3^rd^^−^ and 4^th^-generation cephalosporins of a total of 237 *E. coli* isolates from stages of integrated broiler production were predominantly ≤ 0.5 µg/mL; however, similar to the prevalence of ceftiofur-resistant *E. coli* isolates, the MIC_50_ values for 3^rd^^−^ and 4^th^-generation cephalosporins of *E. coli* isolates from dust prior to placement in broiler breeder farms that practice contract hatching were found to be high at 2–8 µg/mL. In particular, the MIC_50_ values for ceftiofur of *E. coli* isolates from fluff and eggshells from commercial broiler hatcheries of operation A were 8–16 µg/mL, indicating an MIC for resistance.

**Table 4:** **Distribution of minimal inhibitory concentrations for 3**^**rd**^** and 4**^**th**^**-order cephalosporins in** ***E. coli*** **isolated from the production stages of three integrated broiler operations**.

Production stage	Sample type	Distribution of MIC_50_ value (µg/mL) by operation											
		Cefotaxime (3^rd^)			Ceftazidime (3^rd^)			Ceftiofur (3^rd^)			Cefepime (4^th^)		
		A (*n* = 342)^1^	B (*n* = 223)	C (*n* = 237)	A (*n* = 342)	B (*n* = 223)	C (*n* = 237)	A (*n* = 342)	B (*n* = 223)	C (*n* = 237)	A (*n* = 342)	B (*n* = 223)	C (*n* = 237)
Breeder hatchery	Fluff	NT^2^	≤ 0.5	≤ 0.5	NT	≤ 0.5	≤ 0.5	NT	1	≤ 0.5	NT	≤ 0.5	≤ 0.5
	Eggshell	NT	≤ 0.5	≤ 0.5	NT	≤ 0.5	≤ 0.5	NT	1	1	NT	≤ 0.5	≤ 0.5
Broiler breeder farm	Dust prior to placement	8	≤ 0.5	≤ 0.5	2	≤ 0.5	≤ 0.5	8	≤ 0.5	≤ 0.5	4	≤ 0.5	≤ 0.5
	Dust at 25–30 weeks old	≤ 0.5	≤ 0.5	≤ 0.5	≤ 0.5	≤ 0.5	≤ 0.5	≤ 0.5	≤ 0.5	≤ 0.5	≤ 0.5	≤ 0.5	≤ 0.5
	Dust over 50 weeks old	≤ 0.5	≤ 0.5	≤ 0.5	≤ 0.5	≤ 0.5	≤ 0.5	≤ 0.5	≤ 0.5	≤ 0.5	≤ 0.5	≤ 0.5	≤ 0.5
Commercial broiler hatchery	Fluff	≤ 0.5	≤ 0.5	≤ 0.5	2	≤ 0.5	≤ 0.5	16	≤ 0.5	≤ 0.5	4	≤ 0.5	≤ 0.5
	Eggshell	≤ 0.5	≤ 0.5	≤ 0.5	2	≤ 0.5	≤ 0.5	8	≤ 0.5	≤ 0.5	4	≤ 0.5	≤ 0.5
Commercial broiler farm	Dust prior to placement	≤ 0.5	≤ 0.5	≤ 0.5	≤ 0.5	≤ 0.5	≤ 0.5	≤ 0.5	≤ 0.5	≤ 0.5	≤ 0.5	≤ 0.5	≤ 0.5
	Dust at the time of depletion	≤ 0.5	≤ 0.5	≤ 0.5	≤ 0.5	≤ 0.5	≤ 0.5	1	1	1	≤ 0.5	≤ 0.5	≤ 0.5

^1^No. of *E. coli* isolated from the production stages of three integrated broiler operations

^2^NT, not tested

The gray fields indicate the minimum inhibitory concentration values for resistance according to the guidelines of the Clinical and Laboratory Standards Institute breakpoints

### Comparison of antimicrobial resistance

A comparison of antimicrobial resistance between ceftiofur-susceptible and ceftiofur-resistant *E. coli* isolates from the stages of integrated broiler production is shown in Table 5. Compared with the ceftiofur-susceptible *E. coli* isolates, the ceftiofur-resistant *E. coli* isolates presented a greater rate of coresistance to most antimicrobial agents. In particular, the prevalence of resistance to all antimicrobial agents tested except for ciprofloxacin, tetracycline, and trimethoprim-sulfamethoxazole was significantly greater in the ceftiofur-resistant *E. coli* isolates (22.6%–100.0%) than in the ceftiofur-susceptible *E. coli* isolates (0.0%–75.7%) (*p* < 0.05). Moreover, the prevalence of MDR in the ceftiofur-resistant *E. coli* isolates was 96.6%, which was also significantly greater than that in the ceftiofur-susceptible *E. coli* isolates (67.7%) (*p* < 0.05).

**Table 5:** **Comparison of antimicrobial resistance between ceftiofur-susceptible and ceftiofur-resistant** ***E. coli strains* isolated from the production stages of three integrated broiler operations**.

Antimicrobial agent	No. (%) of antimicrobial-resistant *E. coli* isolate by operation							
	Ceftiofur-susceptible *E. coli* isolate				Ceftiofur-resistant *E. coli* isolate			
	A (*n* = 225)^1^	B (*n* = 150)	C (*n* = 188)	Total (*n* = 563)	A (*n* = 117)	B (*n* = 73)	C (*n* = 49)	Total (*n* = 239)
Ampicillin	162 (72.0)_b_^B^	97 (64.7)_b_^B^	167 (88.8)_a_^B^	426 (75.7)^B^	117 (100.0)^A^	73 (100.0)^A^	49 (100.0)^A^	239 (100.0)^A^
Amoxicillin-clavulanate	16 (7.1)	26 (17.3)^B^	42 (22.3)^B^	84 (14.9)^B^	15 (12.8)_c_	38 (52.1)_a_^A^	17 (34.7)_b_^A^	70 (29.3)^A^
Cefoxitin	3 (1.3)^B^	4 (2.7)^B^	3 (1.6)^B^	10 (1.8)^B^	31 (26.5)^A^	18 (24.7)^A^	13 (26.5)^A^	62 (25.9)^A^
Cefotaxime	0 (0.0)^B^	0 (0.0)^B^	0 (0.0)^B^	0 (0.0)^B^	108 (92.3)_a_^A^	58 (79.5)_b_^A^	43 (87.8)_a,b_^A^	209 (87.4)^A^
Ceftazidime	0 (0.0)^B^	0 (0.0)^B^	0 (0.0)^B^	0 (0.0)^B^	60 (51.3)^A^	42 (57.5)^A^	23 (46.9)^A^	125 (52.3)^A^
Cefepime	0 (0.0)^B^	0 (0.0)^B^	0 (0.0)^B^	0 (0.0)^B^	73 (62.4)_a_^A^	34 (46.6)_b_^A^	34 (69.4)_a_^A^	141 (59.0)^A^
Chloramphenicol	93 (41.3)^B^	56 (37.3)^B^	71 (37.8)^B^	220 (39.1)^B^	75 (64.1)^A^	37 (50.7)^A^	25 (51.0)^A^	137 (57.3)^A^
Ciprofloxacin	144 (64.0)_b_	75 (50.0)_c_	162 (86.2)_a_	381 (67.7)	85 (72.6)_b_	38 (52.1)_c_	47 (95.9)_a_	170 (71.1)
Gentamicin	24 (10.7)	7 (4.7)^B^	11 (5.9)^B^	42 (7.5)^B^	22 (18.8)	18 (24.7)^A^	9 (18.4)^A^	49 (20.5)^A^
Tetracycline	144 (64.0)	83 (55.3)	130 (69.1)	357 (63.4)	80 (68.4)	44 (60.3)	33 (67.3)	157 (65.7)
Trimethoprim/sulfamethoxazole	70 (31.1)	41 (27.3)	72 (38.3)^B^	183 (32.5)	35 (29.9)_b_	24 (32.9)_b_	30 (61.2)_a_^A^	89 (37.2)
Meropenem	0 (0.0)	0 (0.0)	0 (0.0)	0 (0.0)	2 (1.7)	0 (0.0)	0 (0.0)	2 (0.8)
MDR	155 (68.9)_a,b_^B^	84 (56.0)_b_^B^	142 (75.5)_a_^B^	381 (67.7)^B^	114 (97.4)^A^	69 (94.5)^A^	49 (100.0)^A^	232 (97.1)^A^

^1^n = No. of *E. coli strains* isolated from the production stages of three integrated broiler operations

The uppercase letters (^A, B^) represent significant differences between ceftiofur-susceptible *E. coli* isolates and ceftiofur-resistant *E. coli* isolates, whereas the lowercase letters (_a, b_) represent significant differences between each operation (*p* < 0.05)

### Distribution of β-lactamase genes by stage of integrated broiler production

The distribution of ceftiofur-resistant *E. coli* isolates harboring the β-lactamase gene by stage of integrated broiler production is shown in Table 6. A total of 222 (92.9%) of the 239 isolates harboured β-lactamase genes, and 166 (69.5%) isolates harboured four types of ESBL β-lactamase genes (*bla*_CTX-M-27_, *bla*_CTX-M-55,_* bla*_CTX-M-65,_ and *bla*_SHV-12_). In particular, *bla*_CTX-M-55_ (6.7%–83.3%) was the most significantly prevalent ESBL gene at all stages of integrated broiler production operations (*p* < 0.05). Moreover, a total of 27 (11.3%) isolates harboured *bla*_CMY-2_, a pAmpC gene. However, the distributions of *bla*_CMY-2_ and *bla*_CTX-M-55_ were significantly different among the three integrated broiler operations (*p* < 0.05). Interestingly, the prevalence rates of *bla*_CMY-2_ and *bla*_CTX-M-55_ in breeder hatcheries of operation B were 93.3% and 6.7%, respectively, whereas the prevalence rates in breeder hatcheries of operation C were the opposite, with prevalences of 25.0% and 75.0%, respectively. Moreover, only six (2.5%) isolates harboured a combination of the ESBL gene and pAmpC gene (*bla*_CTX-M-55_ and *bla*_CMY-2_). Additionally, among non-ESBL β-lactamase genes, *bla*_TEM-1_ was the only gene detected in all stages of integrated broiler production operations, ranging from 45.0% to 83.3%.

**Table 6:** **Distribution of ceftiofur-resistant** ***E. coli* isolates harboring the β-lactamase gene by production stage in three integrated broiler operations**.

Production stage	Operation	No. (%) of isolates carrying each gene (%)							No. (%) of ESBL gene combination of pAmpC gene	No. (%) of *E. coli* isolates harboring one or more β-lactamase gene
		ESBL gene					pAmpC gene	Non-ESBL gene		
		*bla*_CTX-M-27_	*bla*_CTX-M-55_	*bla*_CTX-M-65_	*bla*_SHV-12_	Total	*bla*_CMY-2_	*bla*_TEM-1_	*bla*_CTX-M-55_/*bla*_CMY-2_	
Breeder hatchery	A (NT)^1^	–	–	–	–	–	–	–		–
	B (*n* = 15)^2^	0 (0.0)^B^	1 (6.7)_b_^A^	0 (0.0)^B^	0 (0.0)^B^	1 (6.7)_b_	14 (93.3)_a_	8 (53.3)_b_	0 (0.0)	15 (100.0)
	C (*n* = 12)	0 (0.0)^B^	9 (75.0)_a_^A^	0 (0.0)^B^	0 (0.0)^B^	9 (75.0)_a_	3 (25.0)_b_	8 (66.7)_a_	0 (0.0)	12 (100.0)
	Total (*n* = 27)	0 (0.0)^B^	10 (37.0)^A^	0 (0.0)^B^	0 (0.0)^B^	10 (37.0)	17 (63.0)	16 (59.3)	0 (0.0)	27 (100.0)
Broiler breeder farm	A (*n* = 40)	0 (0.0)_b_^C^	21 (52.5)^A^	4 (10.0)_a,b_^B^	0 (0.0)^C^	25 (63.0)_b_	2 (5.0)_a,b_	18 (45.0)	1 (2.5)	38 (95.0)
	B (*n* = 33)	2 (6.1)_a_^C^	17 (51.5)^A^	7 (21.2)_a_^B^	0 (0.0)^C^	26 (78.8)_a_	3 (9.1)_a_	20 (60.6)	2 (6.1)	31 (93.9)
	C (*n* = 12)	0 (0.0)_b_^C^	6 (50.0)^A^	1 (8.3)_b_^B^	0 (0.0)^C^	7 (58.3)_b_	0 (0.0)_b_	7 (58.3)	0 (0.0)	10 (83.3)
	Total (*n* = 85)	2 (2.4)^C^	44 (51.8)^A^	12 (14.1)^B^	0 (0.0)^C^	58 (68.2)	5 (5.9)	45 (52.9)	3 (3.5)	79 (92.9)
Commercial broiler hatchery	A (*n* = 63)	0 (0.0)_b_^B^	50 (79.4)_a_^A^	1 (1.6)^B^	1 (1.6)^B^	52 (82.5)_b_	4 (6.3)_a_	29 (46.0)_b_	3 (4.8)	59 (93.7)
	B (*n* = 12)	1 (8.3)_a_^B^	10 (83.3)_a_^A^	1 (8.3)^B^	0 (0.0)^C^	12 (100.0)_a_	0 (0.0)_b_	6 (50.0)_b_	0 (0.0)	12 (100.0)
	C (*n* = 12)	0 (0.0)_b_^C^	5 (41.7)_b_^A^	1 (8.3)^B^	0 (0.0)^C^	6 (50.0)_c_	1 (8.3)_a_	10 (83.3)_a_	0 (0.0)	11 (91.7)
	Total (*n* = 87)	1 (1.1)^B^	65 (74.7)^A^	3 (3.4)^B^	1 (1.2)^B^	70 (80.5)	5 (5.7)	45 (51.7)	3 (3.4)	82 (94.3)
Commercial broiler farm	A (*n* = 14)	0 (0.0)^C^	6 (42.9)_b_^A^	1 (7.1)^B^	0 (0.0)^C^	7 (50.0)_b_	0 (0.0)	9 (50.0)_b_	0 (0.0)	12 (85.7)
	B (*n* = 13)	0 (0.0)^C^	9 (69.2)_a_^A^	1 (7.7)^B^	0 (0.0)^C^	10 (76.9)_a_	0 (0.0)	9 (69.2)_a_	0 (0.0)	11 (84.6)
	C (*n* = 13)	0 (0.0)^C^	9 (69.2)_a_^A^	2 (15.4)^B^	0 (0.0)^C^	11 (84.6)_a_	0 (0.0)	7 (53.8)_b_	0 (0.0)	11 (84.6)
	Total (*n* = 40)	0 (0.0)^C^	24 (60.0)^A^	4 (10.0)^B^	0 (0.0)^C^	28 (70.0)	0 (0.0)	25 (62.5)	0 (0.0)	34 (85.0)
Total (*n* = 239)		3 (1.3)^C^	143 (59.8)^A^	19 (7.9)^B^	1 (0.4)^C^	166 (69.5)	27 (11.3)	129 (54.0)	6 (2.5)	222 (92.9)

^1^NT, not tested

^2^n = No. of ceftiofur-resistant *E. coli* isolates

The uppercase letters (A, B) indicate significant differences among the extended-spectrum β-lactamase genes, whereas the lowercase letters (a, b) indicate significant differences among the operations for each β-lactamase gene (*p* < 0.05)

### Distribution of the plasmid replicon types by stage of integrated broiler production

**Figure 3:**
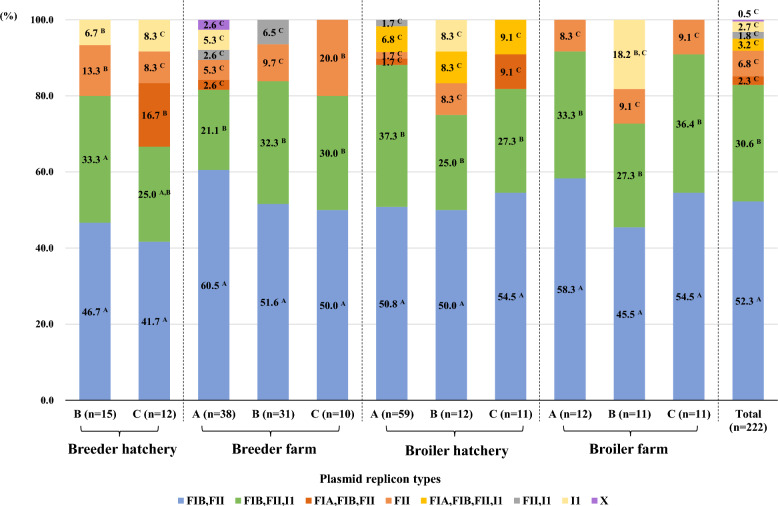
**Distribution of the plasmid replicon types of 222 ceftiofur-resistant *E. coli* isolates harboring β-lactamase genes. **The uppercase letters (^A, B^) represent significant differences among plasmid replicon types in each operation (*p* < 0.05).

The distribution of the plasmid replicon types of 222 ceftiofur-resistant *E. coli* isolates harboring β-lactamase genes is shown in Figure 3. All the isolates harboured at least one or more plasmid replicons, such as IncFIA, IncFIB, IncFII, IncI1, or IncX. Interestingly, regardless of operation, the most significantly prevalent plasmid replicon type in all stages of integrated broiler production operations was a combination of IncFII and IncFIB (41.7%–60.5%), followed by multiple combinations of IncFIB, IncFII, and IncI1 (21.1%–37.3%) (*p* < 0.05).

## Discussion

The presence of ceftiofur-resistant *E. coli* in chicken carcasses from slaughterhouses has been reported in many countries [27–29], and these bacteria pose a significant risk to public health, as they can be transmitted to humans through the consumption of undercooked or contaminated food products [30]. In Korea, the presence of ceftiofur-resistant *E. coli* in chicken carcasses has also been consistently reported [19, 20, 31], and as previously reported [4, 32], the dissemination of ceftiofur-resistant *E. coli* in carcasses may be related to the use of ceftiofur in broiler breeder hatcheries. In this study, the use of ceftiofur at various stages of broiler production was investigated for the first time. In Korea, ceftiofur has been coadministered with only Marek’s disease vaccine in breeder hatcheries and has never been used in broiler breeder farms, commercial broiler hatcheries, or commercial broiler farms. However, in this study, ceftiofur-resistant *E. coli* isolates were identified throughout the stages of integrated broiler production. Cox et al. and Nilsson et al. reported that antimicrobial-resistant *E. coli* can be vertically transmitted from parent flocks to the next generation via fecal contamination of eggshells [33, 34]. Moreover, contact with poultry transport vehicles and visits by transport personnel operating in broiler production systems increase the risk of disease and antimicrobial-resistant bacteria transmission [35, 36]. Therefore, the high presence of ceftiofur-resistant *E. coli* at all stages of integrated broiler production is presumed to have been strongly influenced by the use of ceftiofur in breeder hatcheries.

In this study, the prevalence of ceftiofur-resistant *E. coli* in fluff and eggshells from hatchers of breeder hatcheries (15.4%–47.4%) and commercial broiler hatcheries (31.6%–51.7%) was generally greater than that in environmental dust from broiler breeder farms (11.5%–38.5%) and commercial broiler farms (8.7%–28.9%). Several researchers reported that hatcheries have the ability to spread antimicrobial-resistant pathogens through the stages of integrated broiler production because of their central position in the integrated broiler production system [37], and antimicrobial-resistant bacteria originating from parent flocks may spread within the hatchery and further contaminate eggs or newly hatched birds [38]. Mo et al. also reported that the presence of ceftiofur-resistant *E. coli* in previous flocks can persist for more than six months in the environment, which may have affected the prevalence of ceftiofur-resistant *E. coli* in subsequent flocks [35]. Therefore, the continued use of ceftiofur in breeder hatcheries may be a crucial factor in the environmental contamination of ceftiofur-resistant bacteria at all stages of integrated broiler production and ultimately in their spread to slaughterhouses and carcasses.

Interestingly, in this study, the prevalence of ceftiofur-resistant *E. coli* isolates in dust prior to placement in broiler breeder farms (60.6%) and in fluff and eggshells from commercial broiler hatcheries (51.6%–51.7%) in operation A was significantly greater than that in operations B (28.9%–46.2%) and C (12.5%–37.5%). Biosecurity measures, including cleaning and disinfection, play crucial roles in controlling the presence and dissemination of pathogens [36]. In particular, ESBL/pAmpC-producing *E. coli* are highly likely to survive in areas where cleaning and disinfection are inadequate or not performed [39]. Moreover, ESBL/pAmpC-producing *E. coli* can still be present in the environment even after adequate cleaning and disinfection [40]. In this study, although the broiler breeder of operation A conducted contract hatching, which precluded the assessment of the prevalence of ceftiofur-resistant *E. coli* at the breeder hatchery, environmental dust prior to placement at broiler breeder farms was already found to be highly contaminated with ceftiofur-resistant *E. coli*; therefore, our results indicate that cleaning and disinfection programs must be strictly applied between flocks, especially in operation A.

Interestingly, in this study, the prevalence of cefotaxime-resistant *E. coli* in carcasses from slaughterhouses was ultimately shown to be influenced by the prevalence of ceftiofur-resistant *E. coli* in commercial broiler farms. Specifically, the prevalence of ceftiofur-resistant *E. coli* was significantly greater in breeder hatcheries, broiler breeder farms and commercial broiler hatcheries of operations A and B than in those of C, but the prevalence in dust at the time of depletion in commercial broiler farms was significantly greater in operations B (28.9%) and C (22.7%) than in operation A (12.7%), which resulted in a greater prevalence of cefotaxime-resistant *E. coli* in carcasses from operations B (30.0%) and C (25.0%) than in those from operation A (16.7%). Although the dissemination of ceftiofur-resistant *E. coli* in the stages of integrated broiler production may result from the use of ceftiofur in breeder hatcheries, these results highlight the need for a strategic approach focused on reducing the transmission of antimicrobial-resistant pathogens to humans by improving infection prevention and control, such as farm practices and biosecurity, water sanitation and immunization, on commercial broiler farms.

In this study, ceftiofur-resistant *E. coli* isolates (20.5%–100.0%) presented significantly higher coresistance rates to most of the tested antimicrobial agents than did the ceftiofur-susceptible *E. coli* isolates (0.0%–75.7%). Moreover, the prevalence of MDR in ceftiofur-resistant *E. coli* (97.1%) was also significantly greater than that in ceftiofur-susceptible *E. coli* (67.7%). These results are consistent with those of other studies, which have shown that ceftiofur-resistant *E. coli* typically exhibit coresistance to many other antimicrobial agents [41–43]. This is because ESBL genes are often located on the same plasmids as genetic determinants conferring resistance to non-β-lactam antimicrobial agents, including aminoglycosides and phenicols [44, 45]. Interestingly, in this study, a total of 222 (92.9%) of the 239 ceftiofur-resistant *E. coli* isolates harboured β-lactamase genes, and 166 (69.5%) isolates harboured four types of ESBL genes (*bla*_CTX-M-27_, *bla*_CTX-M-55,_* bla*_CTX-M-65,_ and *bla*_SHV-12_). Among the ESBL genes, *bla*_CTX-M-55_ (6.7%-83.3%) was the most commonly detected gene in all stages of broiler production. Several researchers have reported that *bla*_CTX-M-55,_ a variant of *bla*_CTX-M-15_ differing by only one amino acid substitution (Ala-77Val), has increased structural stability and catalytic efficiency for extended-spectrum cephalosporins, resulting in increased resistance to these antimicrobial agents [46, 47], and has also been increasingly reported in poultry in recent years [48–50].

Moreover, the pAmpC genes have a broader spectrum of resistance, including resistance to cephamycins, and are not inhibited by β-lactamase inhibitors, resulting in resistance to most therapeutically available β-lactams [51, 52]. In particular, *bla*_CMY-2_ is the most commonly detected pAmpC gene in *E. coli* from various host species, including broilers [53–55]. Daniels et al. reported that the emergence of *bla*_CMY-2_ has also been generally attributed to the use of ceftiofur [56]. Moreover, several researchers reported that *bla*_CMY-2_ has been detected in combination with ESBL genes, leading to serious challenges due to the limited therapeutic options [57–59]. In this study, only 27 (11.3%) isolates harboured *bla*_CMY-2_, and of these, only six (2.5%) isolates harboured a combination of *bla*_CMY-2_ and *bla*_CTX-M-55_. Interestingly, the prevalence of *bla*_CMY-2_ was 93.3% in breeder hatcheries of operation B, and fortunately, as in the other operations, the prevalence was low in subsequent stages of broiler production. However, these results indicate that *bla*_CMY-2_ may be widely disseminated across the stages of integrated broiler production in Korea. Moreover, *bla*_CMY-2_ is associated with multidrug resistance because it carries resistance determinants for other antimicrobial agents on the same plasmid.

In this study, all isolates carrying β-lactamase genes harbored at least one or more plasmid replicon, which plays an important role in the horizontal transfer of β-lactamase genes among Enterobacteriaceae [60], and 214 (96.4%) isolates harbored the IncF plasmid. IncF plasmids are frequently found in *E. coli* from humans and animals and often carry more than one replicon, which facilitates replication initiation [61]. Moreover, IncF plasmids can integrate a wide range of genes conferring resistance to all major antimicrobial agents, including β-lactams, aminoglycosides, tetracyclines, phenicols, and quinolones [62, 63]. Interestingly, regardless of operation, the most significantly prevalent replicon type in all stages of broiler production was the combination of IncFIB and IncFII (41.7%–60.5%), followed by multiple combinations of IncFIB, IncFII, and IncI1 (21.1%–37.3%). These results are consistent with other studies showing that the combination of IncFIB and IncFII was the most common replicon type in MDR *E. coli* from broilers and humans [64–66]. Musicha et al. also reported that the combination of IncFII and IncFIB plays a key role in harboring and disseminating antimicrobial resistance genes, including ESBL genes [67]. Therefore, these results suggest that plasmids harboring a wide range of antimicrobial resistance genes can be continuously disseminated throughout the stages of integrated broiler production, and if these plasmids are transmitted to humans, the effectiveness of antimicrobial treatments for bacterial infections may be limited.

This study demonstrated that the use of ceftiofur in breeder hatcheries has a significant effect on the prevalence of ceftiofur-resistant isolates throughout the stages of integrated broiler production. In many countries, including Canada, Japan, the European Union, and the United States, the prevalence of ceftiofur-resistant bacteria in chicken carcasses has decreased since the off-label use of ceftiofur in broiler production stages was prohibited [11–14]. Therefore, in Korea, the off-label use of ceftiofur in breeder hatcheries must also be prohibited either voluntarily or mandatorily to prevent the dissemination of ceftiofur-resistant *E. coli* throughout the stages of integrated broiler production.

## Data Availability

The datasets used and analysed during the current study are available from the corresponding author upon reasonable request.
